# Phosphorus-nitrogen compounds (Part 51): the relationship between spectroscopic and crystallographic data of mono- and di- *spiro* cyclophosphazene derivatives with 4-fluoro/nitrophenylmethyl pendant arm/arms

**DOI:** 10.3906/kim-1911-43

**Published:** 2020-06-01

**Authors:** Aytuğ OKUMUŞ, Gamze ELMAS, Nuran ASMAFİLİZ, Selen BİLGE KOÇAK, Zeynel KILIÇ

**Affiliations:** 1 Department of Chemistry, Faculty of Science, Ankara University, Ankara Turkey

**Keywords:** *spiro* Cyclophosphazene, 4-fluoro/nitrophenylmethyl pendant arm, ^31^P NMR, X-ray crystallography, electron density transfer parameter

## Abstract

A great wealth of structural information about phosphazenes can be gleaned from the combined spectroscopic and crystallographic data. When data from ^31^P NMR spectroscopy and X-ray crystallography are put together like pieces in a puzzle, a number of correlations can be obtained for phosphazene derivatives. A systematic study concerning the correlations among the structural parameters (e.g., ^31^P NMR data, endocyclic/exocyclic NPN bond angles and bond lengths) revealed some characteristics of mono- and di- *spiro* cyclophosphazene derivatives bearing 4-fluoro/nitrophenylmethyl pendant arm/arms. These correlations include the relationship between the δ P_*spiro*_ shifts, the values of electron density transfer parameters Δ(P–N), and the endocyclic and exocyclic NPN bond angles of the cyclophosphazenes. The structural parameters were compared with each other for 19 compounds of 5 different architectural types of cyclophosphazenes with 5- to 7-membered *spiro* -rings.

## 1. Introduction

Since 1960, the Cl-replacement reactions of hexachlorocyclotriphosphazene (trimer, N_3_P_3_Cl_6_) and octachlorocyclotetraphosphazene (tetramer, N_4_P_4_Cl_8_) with monodentate [1,2] and bidentate [3] reagents have been extensively studied. The sequential substitution reactions of the Cl-atoms of N_3_P_3_Cl_6_ and N_4_P_4_Cl_8_ with primary and secondary amines led to the formation of the partly and fully substituted organocyclophosphazenes [4]. The condensation reactions of N_3_P_3_Cl_6_ with bidentate reagents yield some interesting products; e.g., *spiro* -, *ansa-* ,, di *spiro* -, tri *spiro* -, *spiro* - *ansa-* ,, *spiro* - *ansa-* , *spiro* , and *spiro* - *bino-* , *spiro* -cyclotriphosphazenes [5]. In addition to these compounds, tetramer also gives 2,4- *ansa-* ,, 2,4-di *spiro* -, 2,6-di *spiro* - and tetra *spiro* cyclotetraphosphazenes with bidentate reagents [6]. The chlorophosphazenes, N_3_P_3_Cl_6_ and N_4_P_4_Cl_8_ , can undergo regio and stereoselective reactions, as well [7]. In recent years, cyclotri and cyclotetraphosphazenes have started to attract much attention due to their potential stereogenic properties, and biological activities such as antibacterial, antifungal and anti-cancer activities [8–12]. Our group has spent many years on designating and synthesizing novel partly substituted cyclotri and cyclotetraphosphazene derivatives with bidentate ligands {dibenzo-diaza-crown ethers [13–17], dibenzo N_2_O_n_ (n=2–4) [18–21] and benzo NO [22–28] donor type aminopodands, mono- and bis-ferrocenyldiamines [29–37], sodium (ferrocenylmethylamino)-1-alkoxide [38-43]}and multidentate N_2_O_2_ -donor type dibenzo aminopodands [44–46]. Some interesting phosphazene derivatives such as monotopic and ditopic *spiro* -crypta phosphazenes, *spiro* cyclic phosphaza (PNP-lariat) ethers, cyclophosphazenes possessing 6-membered *spiro* ring/rings, mono and bisferrocenyl *spiro* cyclophosphazenes, and *spiro* - *ansa-* , *spiro* -, *spiro* - *bino-* , *spiro* - and *ansa-* , *spiro* - *ansa-* ,phosphazenes were synthesized. Besides this, our research group has long focused on performing substituent exchange reactions of Cl–atoms in partly substituted derivatives with heterocyclic amines {pyrrolidine, piperidine, morpholine, 1,4-dioxa-8-aza *spiro* [4,5]decane, 1-(2-aminoethyl)pyrrolidine, 1-(2-aminoethyl) piperidine, 4-(2-aminoethyl)morpholine}and vanillin side groups, aiming at investigating spectral properties, cytotoxic, antituberculosis and antimicrobial activities, and DNA interactions of the obtained fully substituted cyclophosphazenes. In the last decade, 4-fluorobenzyl pendant armed mono *spiro* and di *spiro* phosphazenes were prepared from the separate reactions of N_3_P_3_Cl_6_ and N_4_P_4_Cl_8_ with 4-fluorobenzyl-NN/NO donor type ligands [47–52]. The phosphezenium salts (protic ionic liquids, PILs or protic molten salts, PMOSs) of fully substituted 4-fluorobenzyl *spiro* cyclotriphosphazenes were also synthesized via reactions of free phosphazene bases with bulky organic acids [53–55]. The spectroscopic and stereogenic properties, and biological activity (antibacterial, antifungal, and cytotoxic activities) of all the (4-fluorobenzyl) *spiro* cyclophosphazenes and some of their phosphazenium salts have been investigated by our research groups [47–55].

Although a large number of papers published by our research group are available on cyclophosphazenes that provide information on their structures, synthesis, and biological activities; the present study focuses on correlation among the structural parameters of mono- and di- *spiro* cyclophosphazene derivatives with 4-fluoro/nitrophenylmethyl pendant arm/arms. In 1986, a systematic study on the relationship between the crystallographic and ^31^P NMR spectral data on phosphazenes was described for the first time by Shaw [56]. Our group has published many studies on the correlations among the structural parameters of various types of cyclotriphosphazenes bearing structurally analogous motifs. It was found out that in cyclotriphosphazene derivatives, variations in the ^31^P NMR shifts depend primarily on the electronic, steric and conformational factors (e.g., electron-releasing and withdrawing powers of substituents, the steric hindrance between the exocyclic groups), and on the differences in the bond lengths and bond angles around the phosphorus atoms, particularly endocyclic (α) and on exocyclic (α′) bond angles. As a particular interest in our ongoing studies on phosphazene-based chemistry, the present study primarily focuses on a number of correlations established among the structural parameters in mono- and di- *spiro* cyclophosphazene derivatives with 4-fluoro/nitrophenylmethyl pendant arm/arms of the compounds previously synthesized and published by our research group (Table 1) [49–52,57–59]. In this context, here we report our findings on the relationship among the δ P_*spiro*_ shifts with endocyclic and exocyclic NPN bond angles, and electron density transfer parameters, and a brief description of the synthesis methods of 5 types and a total of 19 cyclotri/tetraphosphazenes containing 4-fluoro/nitrophenylmethyl pendant arm and 5- to 7-membered *spiro* -rings.

**Table 1:** The endocyclic (α) and exocyclic (α′) NPN bond angles and bond lengths (a, a ′ , b, and b′) on the formulae of cyclophosphazenes.

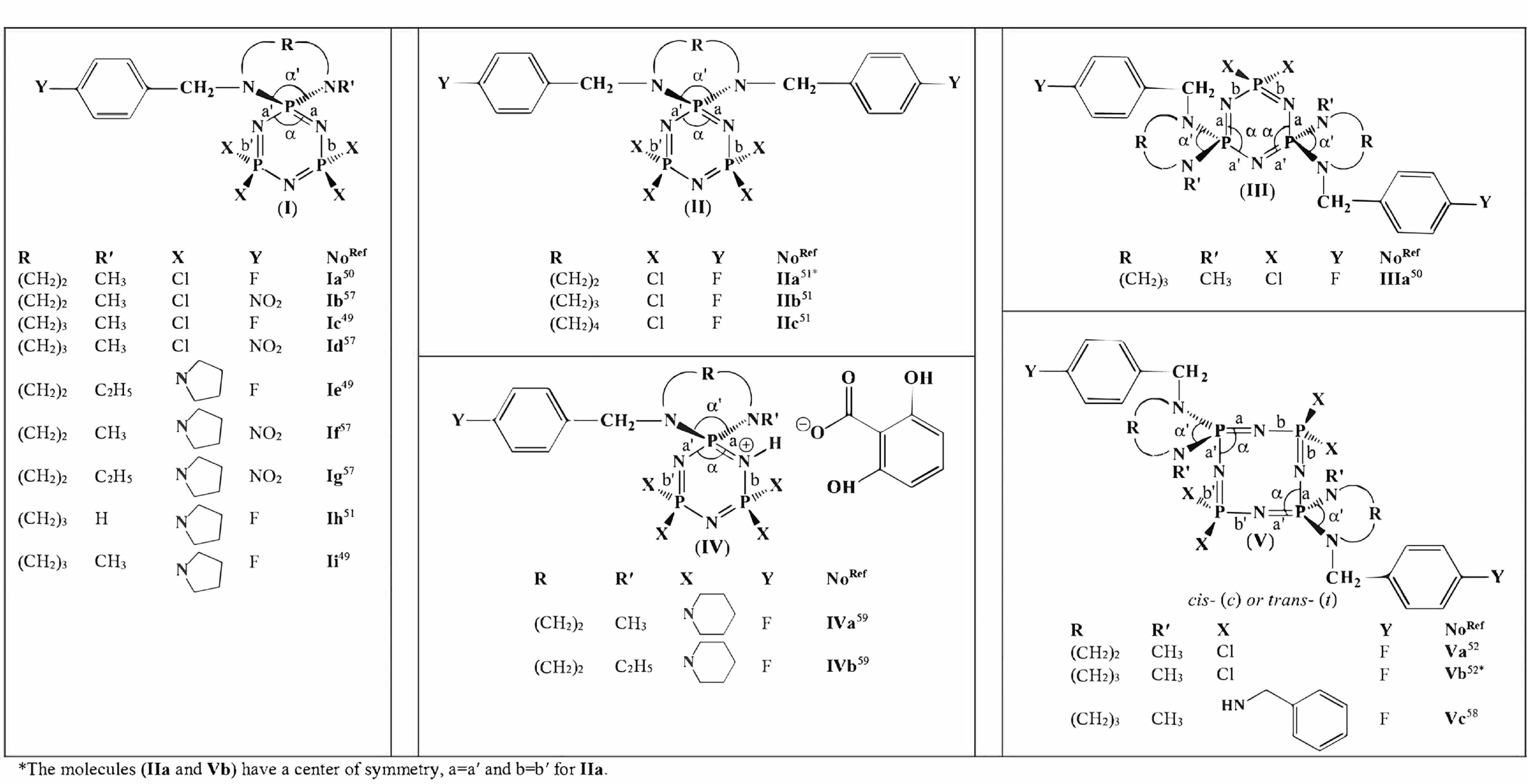

## 2. Results and discussion

### 2.1. Synthesis

Routes used for the preparation of mono- and di- *spiro* cyclophosphazene derivatives with 4-fluoro/nitrophenylmet hyl pendant arm were given in Scheme. *N* -H-R′- *N* ′-mono(4-fluoro/nitrophenylmethyl)diamines [49,57] and bis(4-fluorophenylmethyl)diamines [51] were prepared via reducing the corresponding Schiff bases obtained from the reactions between 4-fluoro/nitrobenzaldehyde and the appropriate *N* -alkyldiamines and *N* , *N* ′- bisalkyldiamines in MeOH. The Cl-replacement reactions of N_3_P_3_Cl_6_ with 4 equimolar amounts of *N* -HR′- *N* ′-mono(4-fluoro/nitrophenylmethyl)diamines in dry THF at ambient temperature to produce 2 different types of products, namely partly substituted mono(4-fluoro/nitrophenylmethyl) *spiro* cyclotriphosphazenes ( **I** ) [49,50,57] and *cis/trans* -bis(4-fluoro/nitrophenylmethyl)di *spiro* cyclotriphosphazenes ( **III** ) [50]. The mono *spiro* ( **I** ) and bisdi *spiro* ( **III** ) derivatives were separated via column chromatography. Partly substituted bis(4- fluorophenylmethyl) *spiro* cyclotriphosphazenes ( **II** ) were synthesized by reacting N_3_P_3_Cl_6_ with bis(4-fluorophen ylmethyl)diamines in dry THF [51]. Fully pyrrolidine substituted phosphazenes ( **I** ) were prepared by replacing 4 Cl-atoms on the partly substituted derivatives ( **I** ) with excess pyrrolidine in boiling THF [49,57]. On the other hand, the partly substituted mono(4-fluorophenylmethyl) *spiro* cyclotetraphosphazenes and *cis/trans* -bis(4- fluorophenylmethyl)di *spiro* cyclotetraphosphazenes ( **V** ) were obtained by reacting N_4_P_4_Cl_8_ with 2 equimolar amounts of *N* -H-R′- *N* ′-mono(4-fluorophenylmethyl)diamines in THF [52]. The 2 different products obtained were separated via column chromatography using toluene. Fully benzylamine substituted bis(4-fluorophenylmeth yl) di *spiro* cyclotetraphosphazene was prepared by reacting partly substituted one with excess benzylamine in dry THF at 25 °C [58]. The PMOS ( **IV** ) derivatives were obtained from the reaction of the corresponding piperidine substituted phosphazenes with gentisic acid in THF [59].

**Scheme:**
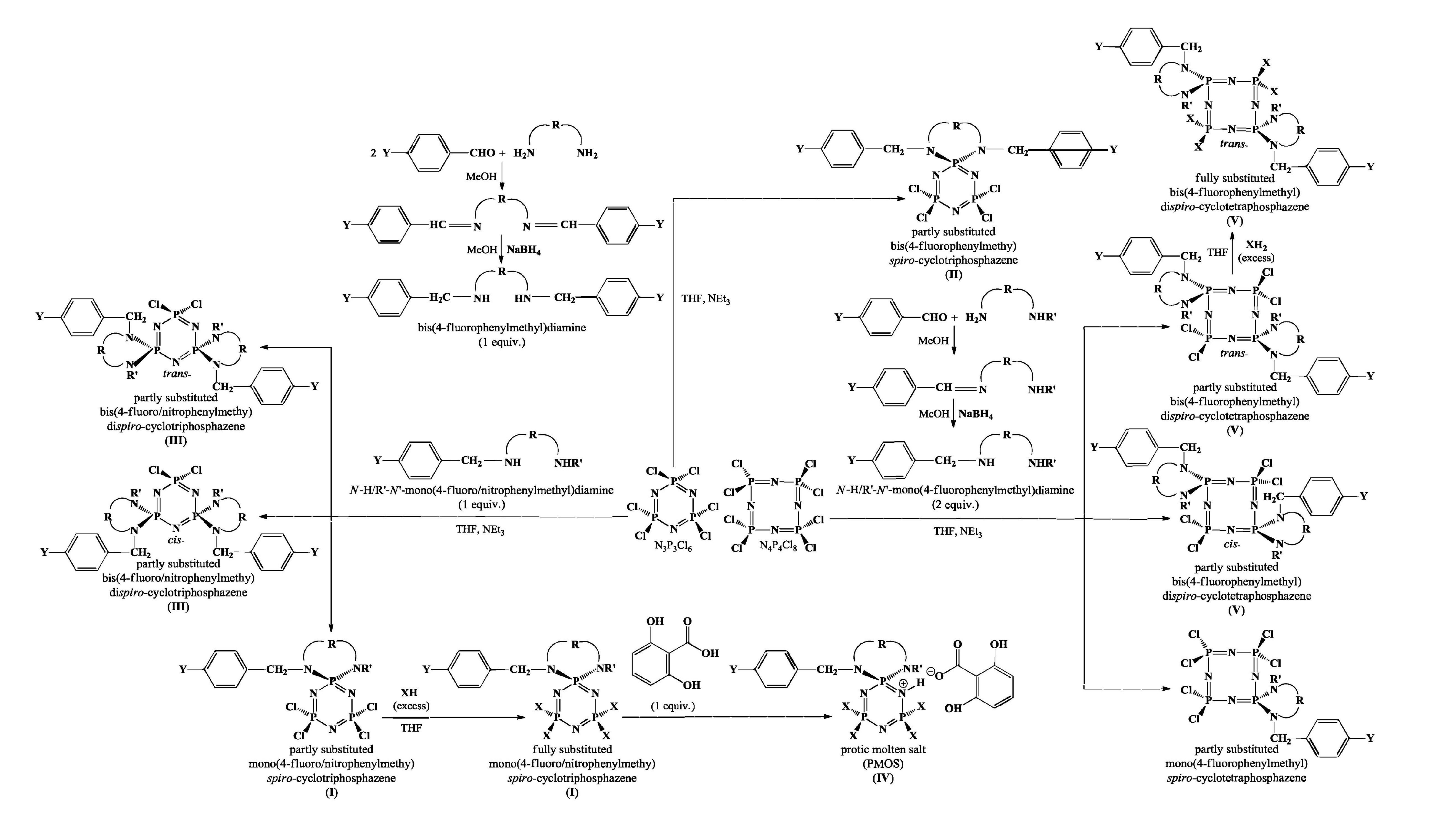
Routes for the preparation of mono- and di-spirocyclophosphazenes with 4-fluoro/nitrophenylmethyl pendant arm/arms investigated in this study.

### 2.2. Correlation among the structural parameters

The endocyclic (α) and exocyclic (α′) NPN bond angles, and the bond lengths (a, a′, b, and b′) on the general formulae of cyclotri/tetraphosphazenes containing 4-fluoro/nitrophenylmethyl pendant arm/arms and 5-, 6- and 7-membered *spiro* -ring/rings are given in Table 1. The δ P_*spiro*_ shifts, α and α′ bond angles, and Δ(P–N) values are listed in Table 2. The corresponding values for the δ P_*spiro*_ shifts of the standard compounds trimer N_3_P_3_Cl_6_ [60,61] and tetramer N_4_P_4_Cl_8_ [62,63] were taken from the literature. Type **I** group members are partly and fully substituted mono(4-fluoro/nitrophenylmethyl) *spiro* -cyclotriphosphazenes. The partly substituted bis(4-fluorohenylmethyl) *spiro* - and di *spiro* -cyclotriphosphazenes constitute type **II** and **III** compounds, respectively. The phosphezenium salts of fully substituted mono(4-fluorophenylmethyl) *spiro* cyclotriphosphazenes are members of type **IV** . Members of type **V** are partly and fully substituted *cis/trans* bis(4-fluorophenylmethyl)di *spiro* cyclotetraphosphazenes.

**Table 2:** Endocyclic (α) and exocyclic (α′) NPN bond angles, bond lengths (a, a′, b, and b′), δP_*spiro*_ shifts and Δ(P-N) values for the cyclophosphazenes [δP_*spiro*_ shifts in ppm, α and α′ angles in °, a, a′, b, and b′ lengths in Å].

Compound	a	a'	b	b'	Δ(P-N)	α	α''	δP_NPN_	
**Ia**^50^	1.607(3)	1.601(3)	1.557(3)	1.555(3)	0.048	111.28(14)	95.46(15)	19.22	for ( **I** ), ( **II** ) and ( **IV** ) and ( **V** ) Δ (P-N)=(a+a')/2-(b+b')/2 for ( **III** ) Δ (P-N)=(a+a')/2-b
	1.607(3)	1.600(3)	1.557(3)	1.556(3)	0.047	111.01(15)	94.97(17)		
**Ib**^57^	1.602(3)	1.614(3)	1.554(3)	1.554(3)	0.054	112.3(1)	95.3(1)	19.35		
**Ic**^49^	1.630(3)	1.607(3)	1.551(3)	1.558(3)	0.064	111.6(1)	103.9(2)	14.34		
**Id**^57^	1.627(2)	1.603(2)	1.559(2)	1.566(2)	0.0525	113.6(7)	103.2(6)	14.35		
**Ie**^49^	1.588(1)	1.590(1)	1.598(1)	1.599(1)	--0.0095	115.1(6)	93.4(5)	27.68		
**If**^57^	1.594(2)	1.592(2)	1.603(2)	1.601(2)	--0.009	115.3(2)	92.11(2)	27.40		
**Ig**^57^	1.589(4)	1.585(4)	1.590(4)	1.603(5)	--0.0095	115.0(2)	94.0(2)	27.25		
**Ih**^51^	1.592(1)	1.592(2)	1.611(1)	1.595(1)	--0.011	118.3(1)	102.4(1)	20.56		
**Ii**^49^	1.595(1)	1.585(1)	1.598(1)	1.606(1)	--0.012	118.2(6)	101.4(6)	23.44		
**IIa**^51*^	1.617(0)	1.617(0)	1.563(1)	1.563(1)	0.0535	111.4(2)	94.7(0)	18.00		
**IIb**^51^	1.631(2)	1.607(2)	1.556(2)	1.560(2)	0.061	111.0(1)	104.2(1)	12.70		
**IIc**^51^	1.619(1)	1.615(2)	1.559(1)	1.561(1)	0.057	113.2(1)	102.6(1)	16.33		
**IIIa**^50^	1.607(2)	1.599(3)	1.566(7)	-	0.037	115.71(12)	104.03(13)	19.98	
	1.630(2)	1.576(3)	1.558(3)	-	0.045	113.90(13)	102.75(13)			
**IVa**^59^	1.651(3)	1.557(3)	1.657(3)	1.607(3)	--0.028	109.86(14)	94.74(14)	13.10		
**IVb**^59^	1.6541(16)	1.5670(17)	1.6634(17)	1.6071(17)	--0.0247	109.86(9)	94.79(10)	13.01		
***t*-Va**^52^	1.588(2)	-	1.542(2)	-	0.046	112.07(9)	99.12(9)	6.54		
***c*-Vb**^52^	1.584(2)	1.611(2)	1.552(2)	1.545(2)	0.049	114.51(12)	102.63(11)	1.52		
***t*-Vb**^52*^	1.555(2)	-	1.570(2)	-	--0.015	112.34(10)	102.14(10)	1.74		
***t*-Vc**^58^	1.5815(18)	1.5851(18)	1.6027(18)	1.5811(18)	--0.0086	118.06(10)	104.10(11)	6.27	
	1.5827(19)	1.5830(18)	1.5970(19)	1.5759(18)	--0.0036	118.11(10)	103.87(10)		

*The molecules ( **IIa** and **Vb** ) have a centre of symmetry, a=a′ and b=b′ for **IIa** IIa.

#### 2.2.1. The relationship among the δ P_*spiro*_ shifts and the electron density transfer parameters Δ(P–N)

**Figure 1:**
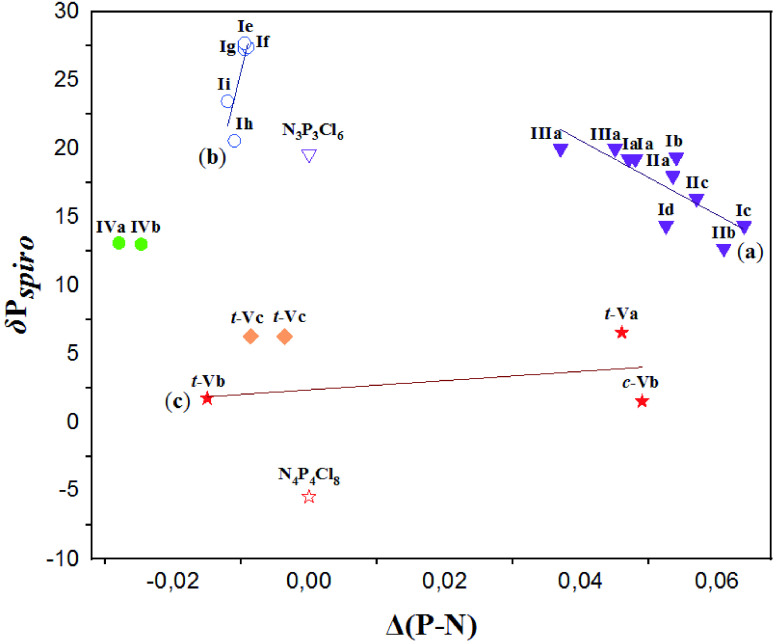
The relationship between δ P_*spiro*_ shifts and Δ(P-N) values for partly and fully pyrrolidine and benzylamine substituted *spiro* cyclic phosphazenes with 4-fluoro/nitrophenylmethyl pendant arm/arms. δ PClPCl shift values of N_3_P_3_Cl_6_ and N_4_P_4_Cl_8_ are 19.60 [61] and –5.45 [63] ppm, respectively.

The electron density transfer parameter Δ(P–N) is the difference between the bond lengths of 2 adjacent endocyclic P-N bonds and is a measure of the electron-releasing and withdrawing powers of the substituents on cyclophosphazene ring. The Δ(P–N) values were calculated using the appropriate equations presented in Table 2 for *spiro* cyclic phosphazenes with 4-fluoro/nitrophenylmethyl pendant arm/arms. If electron-withdrawing substituents are bonded to phosphorus atoms, Δ(P–N) values increase. On the other hand, in case of electronreleasing substituents the Δ(P–N) values decrease. The relationship between the δ P_*spiro*_ shifts and the Δ(P–N) values is given in Figure 1 for partly and fully pyrrolidine and benzylamine substituted *spiro* cyclic phosphazenes.

The linear correlation between δ P_*spiro*_ shifts and Δ(P–N) values observed in 3 groups of cyclophosphazenes are given in Figure 1. When comparing partly substituted types I, II, and III phosphazenes (a) with the fully pyrrolidine substituted type I phosphazenes (b), an inverse relation is observed in Figure 1. The Δ(P–N) values could be interpreted by comparing these values with the ones for partly (a) and fully (b) substituted cyclophosphazenes. While fully pyrrolidine substituted cyclotriphosphazenes (Ie-Ii) have negative Δ(P–N) values, the partly substituted ones (Ia-Id, IIa-IIc and IIIa) have positive values, and the value of the standard compound N_3_P_3_Cl_6_ is zero indicating that the electron-releasing powers of nitrogen atoms in pyrrolidine groups to phosphazene ring is greater than those of the Cl-atoms. Moreover, there is a significant difference between the Δ(P–N) values of cis- and trans-structures of the same compound of type V phosphazenes (c) (0.049 for c-Vb and -0.015 for t-Vb). It is possibly due to the different types of hydrogen bond interactions; e.g., intermolecular C-H—F for t-Vb and intramolecular C-H—N for c-Vb [52]. As expected, the Δ(P–N) value of benzylamine substituted t-Vc is larger than the value of Δ(P–N), which is zero, for the standard compound N_4_P_4_Cl_8_ .

The Y group (F or NO2) placed at the para position on the benzene ring is an electron-withdrawing substituent and does not cause a significant change in the Δ(P–N) values. However, the points of the NO2 - containing compounds (Ib and Id) slightly deviate from the linear trend (Figure 1).

Considering the electron-releasing capacity of the 4-fluorophenylmethyl pendant group for type I-III partly substituted cyclotriphosphazenes with 6-membered *spiro* -ring, the following order is established: IIIa >IIb >Ic. While the compounds Ia and IIb are mono- and bis-4-fluorophenylmethyl *spiro* -structures, respectively, compound IIIa is bis-4-fluorophenylmethyl di- *spiro* structure. As expected, the electron-releasing strength of 2 4-fluorophenylmethyl pendant groups is more than that of 1 4-fluorophenylmethyl pendant group. However, the same trend is not observed for 5-membered Ia and IIa. This is due to the fact that Ia has 2 independent molecules in the asymmetric unit [50].

There is no significant difference between the Δ(P–N) values of type II phosphazenes containing the *spiro* rings with 6- (IIb) and 7- (IIc) membered. However, the Δ(P–N) values of the phosphazene with 6-membered *spiro* -ring (IIa) is slightly larger than that of the phosphazene with 5-membered *spiro* -ring (IIb). That could be significantly attributed to the fact that 5-membered *spiro* -ring of IIa is in the twisted conformation and 6-membered *spiro* -ring of IIb is in the chair conformation [51].

The relationship between Δ(P–N) and δ P_*spiro*_ shifts strongly indicates the basicity of the nitrogen atoms in the phosphazene ring. The basicity of the chlorocyclophosphazene ring containing nitrogen atoms is quite low, and it can be improved by replacing Cl-atoms with electron-releasing substituents on phosphorus. Therefore, the basicity of the nitrogen atoms on the cyclotriphosphazene ring, which is both adjacent (N2- P_*spiro*_) and nonadjacent to the *spiro* -ring (N1-PX2) in fully pyrrolidine substituted cyclotriphosphazenes can be compared with those in partly substituted ones. The basicity of the N1 atom/atoms in fully substituted phosphazenes appear(s) to have increased due to electron-releasing power of the heterocyclic amine groups. However, N2 atoms in partly substituted phosphazenes decreased due to electron-withdrawing power of the Clatoms. Nevertheless, protonation of type I heterocyclic amine substituted free cyclotriphosphazene bases with bulky organic acids (gentisic and γ -resorcylic acids) took place on the N2-atom [49] (type V) instead of N1-atom [54,55] of the 4-fluorobenzyl *spiro* cyclotriphosphazenes. The H+ ion may be exchanged between the N1-and N2-atoms of the cyclotriphosphazene ring in the solution at ambient temperature. The ^31^P NMR spectra recorded at low temperatures and the observed spin-systems in the ^31^P NMR spectra of the PMOSs may also confirm that the H+ ion can be displaced between the nitrogen atoms of the phosphazene ring. Although the number of type V group members is limited, they could be thought of as reference compounds. It appears that the δ P_*spiro*_ shifts and the basicity of the ring decrease after PMOS forms.

The double-bond character of the P-N linkage in the cyclophosphazene derivatives is not fully understood. Negative hyperconjugation and ionic bonding alternatives are exclusive [64]. The natural-bond orbital and topological electron-density analyses of the phosphazenes have proved the crucial role of negative hyperconjugation in description of the P-N bond. An increase in the electron-releasing power of heterocyclic amine substituents seems to cause an increase in the negative hyperconjugation. The electron−withdrawing substituents such as Cl-atom increase the Δ(P–N) values since they attract electrons from *spiro* -ring/rings to the phosphorus atom. However, the electron-releasing substituents such as pyrrolidine group decrease the Δ(P–N) values resulting in decreased bond lengths (a and a′) and increased bond lengths (b and b′) when the bond lengths of partly substituted derivatives are compared. Hence, the decrease in the length of the endocyclic P–N bonds and in electron charge density on the exocyclic P-N bonds are likely to be a measure of the electron-releasing power of the substituent and the increase in negative hyperconjugation.

#### 2.2.2. The relationship among the δ P_*spiro*_ shifts, endocyclic (α), and exocyclic (α′ ) NPN bond angles

**Figure 2:**
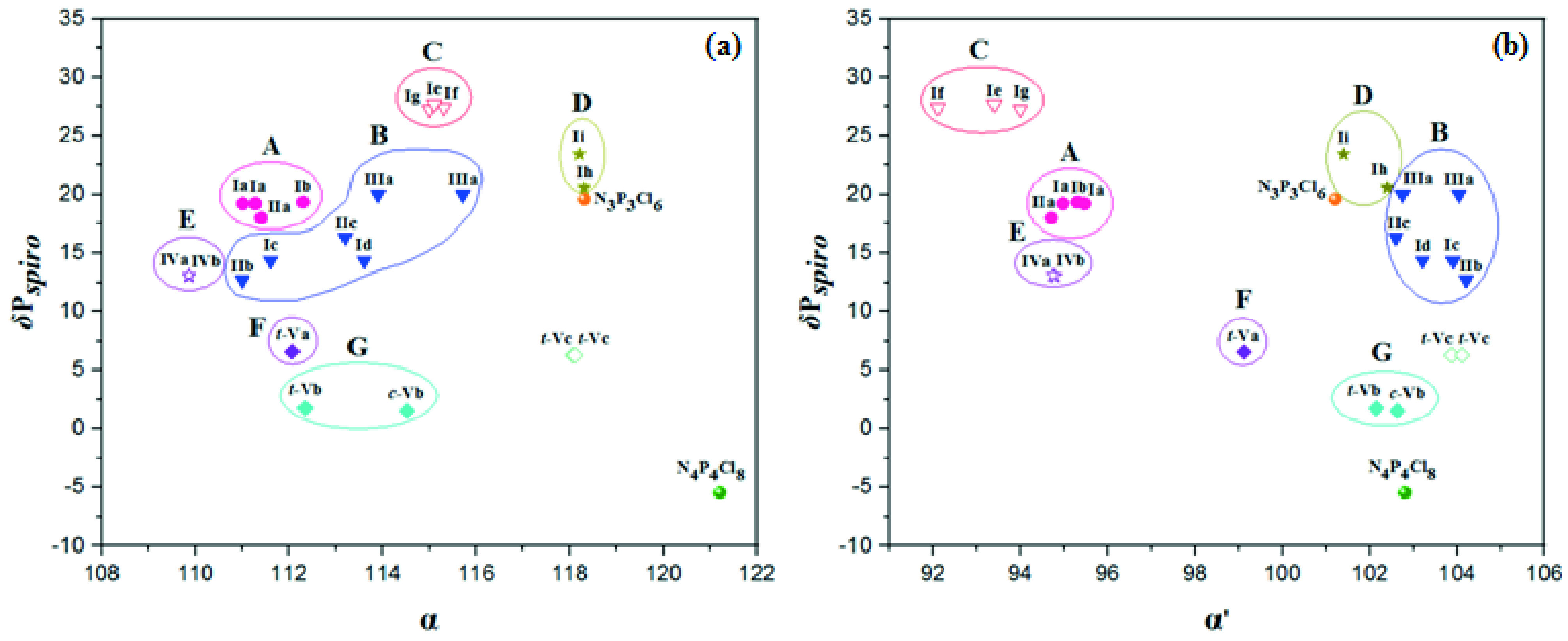
The relationship between δ P_*spiro*_ shifts and endocyclic (α) (a) and exocyclic α′ (b) NPN bond angles for partly and fully pyrrolidine and benzylamine ubstituted *spiro* cyclic phosphazenes with 4-fluoro/nitrophenylmethyl pendant arm/arms. δ PClPCl shift values of N_3_P_3_Cl_6_ and N_4_P_4_Cl_8_ are 19.60 [61] and -5.45 [63] ppm, respectively. The α and α′ values are 118.3(2) and 101.2(1)° for N_3_P_3_Cl_6_ [60], 121.2, and 102.8° for N_4_P_4_Cl_8_ [62], respectively.

A cluster of points rather than the linear trend were observed among the δ P_*spiro*_ shifts, and endocyclic (α) and exocyclic (α′) NPN bond angles. In Figure 2, all types of phosphazene structures were accumulated in 7 regions A, B, C, D, E, F, and G. The points of partly substituted type I-III cyclotriphosphazenes, fully pyrrolidine substituted type I phosphazenes, and partly substituted type V cyclotetraphosphazenes with 5- and 6-membered *spiro* -rings were accumulated in regions (A and B), (C and D), and (F and G), respectively. The points of type IV PMOSs with 5-membered *spiro* -ring were accumulated in region E.

Furthermore, small changes in α and α′ bond angles lead to significant changes in δ P_*spiro*_ shifts. A change in the number of members in the *spiro* -ring causes a major change in both α and α′ bond angles. In fact, the α and α′ bond angles of cyclophosphazenes with 5-membered *spiro* -ring are smaller than those with the 6- and 7-membered ones, and are even smaller than those corresponding to α [118.3(2)°] and α′ [101.2(1)°] bond angles [60] in the standard compound, N_3_P_3_Cl_6_ . Besides, there is a decrease in δ P_*spiro*_ shifts with increasing number of members in the *spiro* -ring. For example, the α′ bond angles of partly substituted Ia with 5-membered *spiro* -ring (δ P_*spiro*_ = 19.22 ppm, cycle A) and Ic with 6-membered *spiro* -ring (δ P_*spiro*_ = 14.34 ppm, cycle B) are respectively; 95.46(15) and 94.97(17), and 103.9(2). This indicates that the electron-releasing power of 5-membered *spiro* -ring to the phosphazene ring is more than that of the 6-membered *spiro* -ring. When partly and fully pyrrolidine substituted type I phosphazenes with the same number of members in the *spiro* -ring (cycles A and C or cycles B and D) are compared, it is seen that the δ P_*spiro*_ shifts increase for fully substituted ones. While the α′ bond angles decrease, the α bond angles increase. This indicates a change in the substituent groups leading to significant changes in both α and α′ bond angles. When considering α bond angles, electrons are transferred from pyrrolidine groups to the cyclotriphosphazene ring in the fully substituted derivatives and from the cyclotriphosphazene ring to Cl-atoms in partly substituted counterparts. When taking into account the α′ bond angles, pyrrolidine groups also release electrons to the phosphazene ring, but the Cl-atoms withdraw electrons not only from the phosphazene ring but also from the *spiro* -ring. The elongation of the 2 exocyclic P–N bonds of the *spiro* -ring is likely the best measure of the electron-withdrawing power of the Cl-atoms and the decrease in negative hyperconjugation.

On the other hand, in tetrameric phosphazenes, the α bond angle of fully benzylamine substituted 6-membered phosphazene (Vc) is larger with respect to the value of partly substituted counterpart (Vb). But, the α′ angle of Vc is larger than the α′ angle of Vb. This situation may be attributed to the basicity or electronreleasing power of benzylamine substituent, which is a secondary aliphatic amine group after the substitution, in Vc not as high as pyrrolidine substituent, a tertiary heterocyclic amine group after the substitution. When compared α and α′ bond angles of type I free phosphazene bases (cycle C) and type IV PMOSs (cycle E) with 5-membered *spiro* -ring, it is observed that the formation of PMOSs of free phosphazene bases results in a decrease in the α bond angles, and increase in the α′ bond angles. In fact, the α′ bond angles of PMOSs (IVa and IVb) are even larger than the corresponding angles in partly substituted cyclotriphosphazenes (cycle A), and the standard compound N_3_P_3_Cl_6_ , indicating that the positive charge on the N2-atom withdraws electrons from the 5-membered *spiro* -ring in PMOSs.

Besides, the α and α′ angles of cis- and trans-structures of the type V cyclotetraphosphazenes with 2 6-membered *spiro* -rings (c-Vb and t-Vb) can be compared with each other. The α and α′ angles of c-Vb are considerably and slightly larger than those of t-Vb, respectively. That could be significantly attributed to the fact that the N4 P4 ring of t -Vb has a twisted conformation and the N4 P4 ring of c-Vb has a boat conformation [52].

## 3. Conclusions

The results of a systematic study of *spiro* -cyclotri/tetraphosphazenes with 4-fluoro/nitrophenylmethyl pendant arm on the basis of correlation between the structural parameters were presented. The main parameters were obtained from X-ray crystallography and ^31^P NMR results in order to investigate the relationship between the δ P_*spiro*_ shift values and endocyclic and exocyclic NPN bond angles, and electron density transfer parameters. The correlations obtained from the present study ought to be considered as highly informative. Although there are visual comparisons for assessing the accuracy of the relationships, more values are required to learn more about the correlations for cyclophosphazenes. In this approach, our research group or one can plot on the same relationships the new values of the other members of mono- and di- *spiro* cyclophosphazene derivatives bearing 4-fluoro/nitrophenylmethyl pendant arm.

## References

[1] (2007). Cyclophosphazene-based multi-site coordination ligands. Coordination Chemistry Reviews.

[2] (2019). Organophosphorus Chemistry.

[3] (2015). Organophosphorus Chemistry.

[4] (2004).

[5] (2009). Phosphorus-nitrogen compounds: Part 16. Journal of Inclusion Phenomena and Macrocyclic Chemistry.

[6] (2014). Phosphorus-nitrogen compounds. Part.

[7] (1991). Regio- and stereochemical control in substitution reactions of cyclophosphazenes. Chemical Reviews.

[8] (2015). Syntheses, structural characterization and biological activities of
*spiro*
-
*ansa-*
,
*spiro*
cyclotriphosphazenes. New Journal of Chemistry.

[9] (2014). Synthesis, characterization and antiproliferative activity of hexa arm starshaped thiosemicarbazones derived from cyclotriphosphazene core. Inorganica Chimica Acta.

[10] (2008). Kumara Swamy KC. Single diastereomers of unsymmetrical tris-
*spiro*
cycliccyclotriphosphazenes based on 1.

[11] (2015). Chiral configurations in cyclophosphazene chemistry. Coordination Chemistry Reviews.

[12] (2017). Phosphorus-nitrogen compounds. Part.

[13] (2006). Phosphorus-nitrogen Compounds: Part 13. Syntheses, crystal structures, spectroscopic, stereogenic and anisochronic properties of novel
*spiro*
-
*ansa-*
,
*spiro*
-,
*spiro*
-
*bino-*
,
*spiro*
- and
*spiro*
-crypta phosphazene derivatives. Inorganic Chemistry.

[14] (2004). Phosphorus-nitrogen compounds: Novel \textit{
*spiro*
}-crypta-phosphazenes. Structure of \textbraceleft pentane-3-oxa-. textit{bis}(1.

[15] (2009). Phosphorus-nitrogen compounds: Part 16. Journal of Inclusion Phenomena and Macrocyclic Chemistry.

[16] (2007). Novel phosphazene derivetives. Synthesis, anisochronism and structural investigations of mono- and ditopic \textit{
*spiro*
}-crypta phosphazenes. Journal of Molecular Structure.

[17] (2008). Phosphorus-nitrogen compounds: Part 15. Journal of Chemical Sciences.

[18] (2010). Phosphorus-nitrogen compounds. Part.

[19] (2005). Phosphorus-nitrogen compounds: Novel \textit{
*spiro*
}-cyclophosphazenic lariat (PNP-pivot) ether derivatives. Structures of 4.

[20] (2005). Phosphorus-nitrogen compounds: Novel fully substituted \textit{
*spiro*
}-cyclophosphazenic lariat (PNP-pivot) ether derivatives. Structures of 4.

[21] (1999). Phosphorus-nitrogen compounds: Part IV. New podand and lariat ether-type macrocycles with cyclophosphazenes. Structure of 2.

[22] (2004). Phosphorus-nitrogen compounds. \textit{
*spiro*
}- and crypta-phosphazene derivatives: Synthesis and spectral investigations. Structure of butane-N,N'-bis(1.

[23] (2005). Phosphorus-nitrogen compounds: New
*spiro*
-cyclic phosphazene derivatives. Structure of 4$^\prime$.

[24] (2007). Phosphorus-nitrogen compounds: Part 14. Inorganic Chemistry.

[25] (2007). Phosphorus-nitrogen compounds: synthesis and spectral investigations on new \textit{
*spiro*
}-cyclic phosphazene derivatives. Spectrochimica Acta Part A: Molecular and Biomolecular Spectroscopy.

[26] (2010). Phosphorus-nitrogen compounds. 21. Syntheses, structural investigations, biological activities, and DNA interactions of new N/O
*spiro*
cyclic phosphazene derivatives. The NMR behaviors of chiral phosphazenes with stereogenic centers upon the addition of chiral solvating agents. Inorganic Chemistry.

[27] (2012). Phosphorus-nitrogen compounds. Part.

[28] (2018). textit{
*spiro*
}-Cylotriphosphazenes containing 4-hydroxyphen ylethyl pendant arm: Syntheses, structural characterization and DNA interaction study. Inorganica Chimica Acta.

[29] (2009). Phosphorus-nitrogen compounds. 18. Syntheses, stereogenic properties, structural and electrochemical investigations, biological activities, and DNA interactions of new
*spiro*
cyclic mono- and bisferrocenylphosphazene derivatives. Inorganic Chemistry.

[30] (2013). Phosphorus-nitrogen compounds: Part 25. Syntheses, spectroscopic, structural and electrochemical investigations, antimicrobial activities, and DNA interactions of ferrocenyldiaminocyclotriphosphazenes. Phosphorus, Sulfur, and Silicon and the Related Elements.

[31] (2013). Phosphorus-nitrogen compounds: Part 26. Syntheses, spectroscopic and structural investigations, biological and cytotoxic activities, and DNA interactions of mono and bisferrocenyl
*spiro*
cyclotriphosphazenes. Inorganica Chimica Acta.

[32] (2013). Phosphorus-nitrogen compounds: Part 28. Syntheses, structural characterizations, antimicrobial and cytotoxic activities, and DNA interactions of new phosphazenes bearing vanillinato and pendant ferrocenyl groups. Journal of Molecular Structure.

[33] (2014). Syntheses of chiral phosphazenes with stereogenic centers: NMR behavior in the presence of a chiral solvating agent. Heteroatom Chemistry.

[34] (2016). Phosphorus-nitrogen compounds. Part.

[35] (2017). Phosphorus-nitrogen compounds. Part.

[36] (2018). Syntheses, spectroscopic and crystallographic characterizations of cis- and trans-di
*spiro*
cyclic ferrocenylphosphazenes: molecular dockings, cytotoxic and antimicrobial activities. New Journal of Chemistry.

[37] (2019). Phosphorus-nitrogen compounds: Part 45. Journal of Molecular Structure.

[38] (2015). Phosphorus-nitrogen compounds: Part 30. Syntheses and structural investigations, antimicrobial and cytotoxic activities and DNA interactions of vanillinato-substituted NN or NO
*spiro*
cyclic monoferrocenyl cyclotriphosphazenes. Journal of Biological Inorganic Chemistry.

[39] (2018). Phosphorus-nitrogen compounds. Part.

[40] (2018). Phosphorus-nitrogen compounds. Part.

[41] (2010). Phosphorus-nitrogen compounds: Part 19. Syntheses, structural and electrochemical investigations, biological activities, and DNA interactions of new
*spiro*
cyclic monoferrocenylcyclotriphosphazenes. Polyhedron.

[42] (2018). Phosphorus-nitrogen compounds: Part 43. Syntheses, spectroscopic characterizations and antimicrobial activities of cis-. Journal of Molecular Structure.

[43] (2019). Phosphorus-nitrogen compounds. Part.

[44] (2004). Phosphorus-nitrogen compounds: Novel
*spiro*
cyclic phosphazene derivatives. Structure of 3.

[45] (2013). Syntheses, spectroscopic properties, crystal structures, biological activities, and DNA interactions of heterocyclic amine substituted \textit{
*spiro*
}-\textit{ansa}-\textit{
*spiro*
}- and \textit{
*spiro*
}-\textit{bino}-\textit{
*spiro*
}-phosphazenes. Inorganica Chimica Acta.

[46] (2015). Syntheses, structural characterization and biological activities of \textit{
*spiro*
-
*ansa-*
,. New Journal of Chemistry.

[47] (2017). The reactions of N$_{3}$P$_{3}$Cl$_{6}$ with monodentate and bidentate ligands: The syntheses and structural characterizations, in vitro antimicrobial activities and DNA interactions of 4-fluorobenzyl(N/O)
*spiro*
cylotriphosphazenes. Turkish Journal of Chemistry.

[48] (2018). Phosphorus-nitrogen compounds. Part.

[49] (2013). Phosphorus-nitrogen compounds. Part.

[50] (2019). The conventional and microwave-assisted syntheses of di
*spiro*
cyclotriphosphazene derivatives with (4-fluoro/4-nitrobenzyl) pendant arms: Structural and stereogenic properties and DNA interactions. Phosphorus-nitrogen Compounds. Part 47.

[51] (2011). Phosphorus-nitrogen compounds. Part.

[52] (2017). Phosphorus-nitrogen compounds. Part.

[53] (2016). Phosphorus-nitrogen compounds. Part.

[54] (2016). The Syntheses and structural characterizations, antimicrobial activity and in vitro DNA binding of 4-fluorobenzylspıro(N/O)cyclotriphosphazenes and their phosphazenium salts. Journal of the Turkish Chemical Society, Section A: Chemistry.

[55] (2017). The spectroscopic and thermal properties, antibacterial and antifungal Activity and DNA interactions of 4-(fluorobenzyl)
*spiro*
(N/O)cyclotriphosphazenium salts. Journal of the Turkish Chemical Society, Section A: Chemistry.

[56] (1986). The phosphazenes-structural parameters and their relationships to physical and chemical properties. Phosphorus, Sulfur, and Silicon and the Related Elements.

[57] (2016). Phosphorus-nitrogen compounds part 33. In vitro cytotoxic and antimicrobial activities, DNA interactions, syntheses and structural investigations of new mono(4-nitrobenzyl)
*spiro*
cyclotriphosphazenes. Research on Chemical Intermediates.

[58] (2017). The reactions of 2-trans-6-bis(4-fluorobenzyl)
*spiro*
cyclotetraphosphazene with primary amines: Spectroscopic and crystallographic characterizations. Phosphorus, Sulfur, and Silicon and the Related Elements.

[59] (2017). Antiproliferative effects against A549, Hep3B and FL cell lines of cyclotriphosphazene-based novel protic molten salts: Spectroscopic, crystallographic and thermal results. Chemistry Select.

[60] (Theoretical 1971). An improved determination of the crystal structure of hexachlorocyclotriphosphazene (phosphonitrilic chloride). Journal of the Chemical Society A: Inorganic.

[61] (2005). Synthesis and characterization of alkyl- and acyl-substituted oxime-phosphazenes. Canadian Journal of Chemistry.

[62] (1968). The crystal structure of compounds with (N-P)$_{n}$ rings. IV. The stable modification ($T$ form) of tetrameric phosphonitrilic chloride. Crystal Engineering and Materials.

[63] (2009). Formation of novel
*spiro*
,
*spiro*
ansa and di
*spiro*
ansa derivatives of cyclotetraphosphazene from the reactions of polyfunctional amines with octachlorocyclotetraphosphazatetraene. Journal of Chemical Sciences.

[64] (2005). Revisiting the electronic structure of phosphazenes. Inorganic Chemistry.

